# Nanotechnology-Based Cardiac Targeting and Direct Cardiac Reprogramming: The Betrothed

**DOI:** 10.1155/2017/4940397

**Published:** 2017-12-11

**Authors:** Fabiana Passaro, Gianluca Testa, Luigi Ambrosone, Ciro Costagliola, Carlo Gabriele Tocchetti, Francesca di Nezza, Michele Russo, Flora Pirozzi, Pasquale Abete, Tommaso Russo, Domenico Bonaduce

**Affiliations:** ^1^Department of Molecular Medicine and Medical Biotechnologies, University of Naples Federico II, Napoli, Italy; ^2^Interdepartmental Center for Nanotechnology Research-NanoBem, University of Molise, Campobasso, Italy; ^3^Department of Medicine and Health Sciences “Vincenzo Tiberio”, University of Molise, Campobasso, Italy; ^4^Department of Translational Medical Sciences, University of Naples Federico II, Napoli, Italy; ^5^Department of Biosciences and Territory (DIBT), University of Molise, Campobasso, Italy

## Abstract

Cardiovascular diseases represent the first cause of morbidity in Western countries, and chronic heart failure features a significant health care burden in developed countries. Efforts in the attempt of finding new possible strategies for the treatment of CHF yielded several approaches based on the use of stem cells. The discovery of direct cardiac reprogramming has unveiled a new approach to heart regeneration, allowing, at least in principle, the conversion of one differentiated cell type into another without proceeding through a pluripotent intermediate. First developed for cancer treatment, nanotechnology-based approaches have opened new perspectives in many fields of medical research, including cardiovascular research. Nanotechnology could allow the delivery of molecules with specific biological activity at a sustained and controlled rate in heart tissue, in a cell-specific manner. Potentially, all the mediators and structural molecules involved in the fibrotic process could be selectively targeted by nanocarriers, but to date, only few experiences have been made in cardiac research. This review highlights the most prominent concepts that characterize both the field of cardiac reprogramming and a nanomedicine-based approach to cardiovascular diseases, hypothesizing a possible synergy between these two very promising fields of research in the treatment of heart failure.

## 1. Introduction

Cardiovascular diseases represent the first cause of morbidity in Western countries, and, although in recent years substantial strides have been made in treatment strategies, mortality still remains high [1].

In particular, chronic heart failure (CHF) is highly prevalent in the general population worldwide, reaching more than 20% in individuals aged > 80 years representing a significant health care burden. It is commonly the end stage in the cardiovascular disease (CVD) continuum, mainly due to coronary heart disease and hypertension [1, 2]. The outcomes for CHF still remain poor and only few patients access the gold standard treatment and heart transplantation [3].

In the last decade, medical research has focused its efforts on the attempt of finding new possible strategies for the treatment of CHF. Several approaches have been tried and have shown promising preliminary results.

Among these, the regenerative hypothesis and stem cells have gained credits especially after the setup of protocols to reprogram cellular fate to definite phenotypes suitable for regenerative purposes [4]. Nevertheless, the use of integrative viruses, frequently adopted in many reprogramming approaches, generates concern mostly related to their association with the risk of gene damage and neoplastic transformation.

The scientific community is concentrating research efforts to identify biochemical pathways involved in the process of direct cardiac reprogramming, to setup more refined protocols in which the typical and potentially harmful tools are progressively replaced with more safe strategies and compounds. The use of small molecules to induce transdifferentiation via nongenetic strategies might provide substantial foundation to drug-based approaches.

First developed for cancer treatment, nanotechnologies offer new therapeutic perspectives in several medical fields of tissue regeneration [5], especially in the cardiovascular context, where the use of chemical compounds for direct cardiac reprogramming can benefit from a nanotechnology-based approach.

This review highlights the most prominent concepts that characterize both the field of cardiac reprogramming and a nanomedicine-based approach to cardiovascular diseases, hypothesizing a possible synergy between these two very promising fields of research in the treatment of heart failure.

## 2. Nanotechnology in Cardiovascular Diseases

To depict the path that led to the development of nanomedicine, it is necessary to begin with the definition which is literally the “intentional design, characterization, production, and applications of materials, structures, devices, and systems by controlling their size and shape in the nanoscale range (1 to 100 nm)” [6]. The genesis of this concept lies in the ideas of the late Nobel physicist Richard P. Feynman, who, in his celebrated talk in 1959, first suggested that “there is plenty of room at bottom” proposing to “use machine tool to make smaller machine tool to be used in turn to make still smaller machine tool and so on to the atomic level” [7]. This view gave birth to the first known concept of nanomedicine, as at the same time Feynman suggested that “although a little wild, it would be interesting if in surgery you could swallow the surgeon which could find out the faulty heart valve and slices it out.” Nowadays, nanomedicine is simply defined as “the medical application of nanotechnology” [8] and is one of the most promising field of research in biomedical sciences [9]. Nanomedicine relies on the physical properties of materials and on their plasticity to interact with biologic structures at the molecular level holding the possibility of being designed to be functionalized as carriers, monitors, detectors, and deliverers [5]. Many medical applications have stemmed from these features for the diagnosis and treatment of diseases leading to the development of several medical tools; some of them are already used in clinical practice [5].

### 2.1. Nanomedicine

As previously highlighted, one of the most relevant fields of nanotechnology is drug delivery. Besides limited solubility, poor distribution within the body, and unfavorable pharmacokinetics behavior [10], traditional medicine is also characterized by the lack of specificity in recognition and interaction with cells and tissues paving the way to side effects and treatment dropout [10]. Nanotechnology-based drug carriers could allow for the delivery of molecules with biological activity at a sustained and controlled rate in a tissue or even cell-specific manner, maximizing therapeutic effects and minimizing consequences of systemic delivery [10].

The interaction of nanocarriers is tantamount for their targeting to specific cells and tissues [11]. Many of the features of nanomedicine lie in this interaction and, of course, it has to be shaped based on the needs [12]. Thus, the nanocarrier design has to take into account all the parameters involved in its interaction with the structure that needs to be targeted. These parameters are mainly the size, the shape, and the surface charge [11]. The size of the nanoparticle is a fundamental characteristic of the nanocarrier because at the same time it has to be tailored to the target's characteristics in order to facilitate the internalization and to be able to interact with the organism's structure, as for example, blood vessels. These considerations set the higher possible diameter under the 4 micrometers; the best diameter reported to be comprised between 95 and 200 nanometers, depending on the targets [13–15]. The shape of nanocarriers strongly affects its interaction with the target because it influences cell surface binding and the speed of the internalization process, the angle of interaction with the target, and the contact surface area being considered important variables [16, 17]. Nevertheless, several observations point to the fact that spherical nanoparticles have a higher probability of internalization as compared to rod-shaped ones, nanocylindrical, and cubic [11, 14, 18]. The surface charge characteristics of nanocarriers are involved not only in the interaction with the target but also in particle aggregation and in the interaction while in the bloodstream. Current knowledge, derived from a large amount of studies [19–23], is that cationic and neutral nanocarriers have the best internalization efficiency due to the attraction with the negative surface charges of the cell membrane.

Once the nanocarrier interacts with the cell, there are several mechanisms by which the cell can internalize it. The process of pinocytosis encompasses macropinocytosis and clathrin- and caveolae-mediated endocytosis. Macropinocytosis is independent of clathrin and caveolin and allows the internalization of large particles (>1 micrometer) via the formation of a membrane protrusion, due to actin polymerization. Clathrin and caveolin mediate the internalization of the nanoparticle based on the direct interaction with the cell membrane and involve mainly 50–150 nm-sized particles [11, 12]. Another process of endocytosis is phagocytosis which allows the internalization of larger particles like, for example, pathogens. This process starts with the opsonization of the particle and the subsequent involvement of the complement receptors. Finally, the surface properties of nanoparticles are very important to reach the target. Nanocarriers, once entered the body, need to reach the target tissue or organ going through the reticuloendothelial system and, hence, need to be masked to the immune system and to other undesired interactions. Several surface modifications have proved efficient in masking nanocarriers to the immune system or interacting with circulating proteins. The most relevant modifications are based on the use of PEG to create repulsive forces against interactions or on the use of chitosan which interacts with tight junctions to facilitate paracellular transport [11]. Other strategies to precisely target tissues and cells are based on the use of proteins and ligand attachments which improve cellular uptake by stimulating the interaction with specific surface proteins and receptors [11].

## 3. Targeting the Heart with Nanocarriers

Cancer treatment has been the first field, in the early 2000s, to benefit from the use of nanotechnology and in particular from the use of nanocarriers in order to improve the efficiency of the drug delivery minimizing side effects and maximizing treatment efficacy [24]. The overall strategy is based on organ/cell-specific targeting which can be achieved not only by designing nanocarriers with specific features but also taking account of the specific target's characteristics. Cancer targeting has taken advantage of the well-established enhanced permeability and retention (EPR) effect [25]. In 1986, the concept of EPR stemmed from the observation that therapeutic macromolecules distributed and accumulated preferentially into the tumor interstitium, and this effect was attributed to the presence of fenestrations in the imperfect tumor blood vessels and to the poor lymphatic drainage in the tissue. Although subsequent research has continuously characterized the EPR effect with many other features which vary among patients and different types of tumors, increased permeability and retention remain the cornerstones of this phenomenon. Nonetheless, other factors affect the magnitude of the EPR, such as the extracellular matrix and its interaction with the extravasated objects [24].

Hence, while the EPR effect represents the main force driving passive targeting, these latter factors affecting the EPR effect are obstacles to an effective nanocarrier-based approach which can, in turn, become a precious ally for a successful strategy. In fact, active targeting is based on the recognition of a ligand by its target and can be used to ultimately refine tissue and cell specificity of nanocarriers.

### 3.1. Cardiac Passive Targeting

Similarly to cancer research, that took advantage of the tumor's biological characteristics, cardiac passive targeting can also stem from heart anatomy and pathophysiology.

The adult heart is composed of several cell types [26] and almost all of them are involved in the development of fibrosis, the major pathophysiological process that leads to cardiac dysfunction and CHF [27]. Cardiomyocytes (CMs) represent the major cell population of the heart [28], followed by cardiac fibroblasts (CFs), endothelial cells (ECs), and vascular smooth muscle cells (VSMCs) [28]. In the development of fibrosis, these cell populations are flanked by components of the immune system, such as monocytes and macrophages, mast cells, and lymphocytes [26]. It can be argued that during this process in the heart, several different phenomena take place which can be used as driving force for a cardiac EPR effect.

Following myocardial infarction (MI), massive CM death results in replacement with fibrous tissue. This process is complex and is due to a series of events and starts with the activation of immunity and the release of cytokines, chemokine, adhesion molecules, and other vasoactive substances that increase vascular permeability with subsequent infiltration with leucocytes. After a phase in which the infarct site is cleared from dead cells, the inflammatory process leaves space to a proliferative phase mediated by the activation of macrophages and differentiation of myofibroblasts, triggered by angiotensin II, PDGF, TGF*β*, which are responsible for extracellular matrix deposition. At this stage, a rich microvascular network is formed, under the effects of proangiogenic factors, to improve oxygen supply to the healing tissue. At the end of this reparative process, the scar undergoes maturation through the cross-linking of extracellular matrix components. Nevertheless, fibroblasts and inflammatory cells in the infarct border zone and in the remote remodeling myocardium may persist, and pressure and volume loads may provide stimulatory signals to their activation. Once the remodeling process has begun, systolic and diastolic dysfunction are the triggers for the onset of heart failure. During this process, the neurohormonal activation and the circulatory dysfunction lead to the onset of a persistent inflammatory state which has further systemic and local negative effects [29–31].

**Figure 1:**
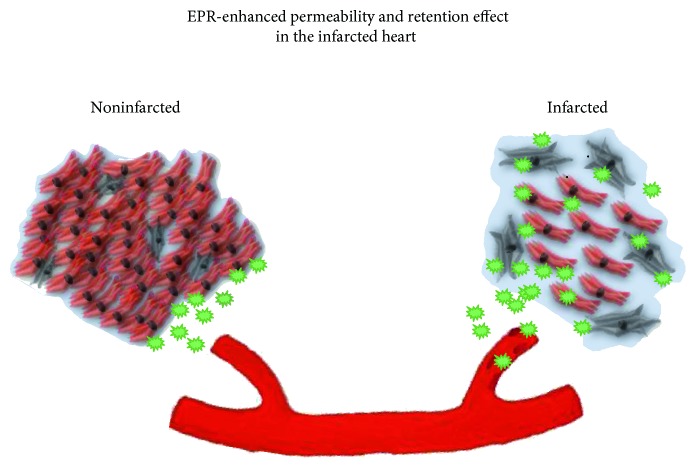
Schematic depiction of the possible EPR effect in the infarcted heart. See text.

All the phases of this process can fulfill the definition of the EPR effect. The infarct site is a place where inflammation and vasoactive mediators alter vascular permeability, and reactive hyperemia can play a role in increased delivery of nanocarriers. Also during the maturation process, the cross-linking of the extracellular matrix components can increase the retention of nanocarriers in the area of interest (Figure 1). A practical application of the infarcted myocardium altered permeability, and retention is exploited in cardiovascular magnetic resonance (CMR) which, as of today, represents a very powerful diagnostic tool in the assessment of myocardial viability [32]. In particular, the tissue characterization of the infarcted myocardium is reliably assessed using the hyperenhancement of gadolinium, the contrast agent used in CMR [33]. This effect consists of the increased enhancement in the infarcted or scarred myocardium due to the gadolinium-increased interstitial space accumulation (wash-in) and reduced clearance (washout) [34]. Several mechanisms have been reported as responsible for this effect: the hyperemia related to the increased vascular permeability, microvascular flow reduction, cardiomyocytes loss, and alteration of regional electrolyte concentrations [35, 32].

The altered permeability and retention exploited in CMR might represent a drive for cardiac passive homing of nanoparticles. Moreover, the experiences achieved in CMR may help improvements in cardiac nanoparticle design.

### 3.2. Cardiac Active Targeting

Active targeting is based on the recognition of a ligand by its target.

Potentially, all the cellular components of the heart and coronary vessels and all the mediators and structural molecules involved in the fibrotic process can be selectively targeted by nanocarriers.

Vascular targeting has extensively been studied for cancer nanotargeting [24]. In cardiac research, instead, only few experiences have been made. To our knowledge, in the heart, the following components have been targeted: the whole infarcted area, cells composing vessels, and cells involved in the postinfarction inflammation process. In 2005, 4 heart endothelial cell-targeting peptides and their receptors were identified [36, 37]. Among these, the CRPPR peptide displayed a marked specificity for cardiac endothelium (300-fold greater than other organs). The proteins selectively expressed by the heart endothelium were in most cases also expressed by CMs and, at lower levels, in some other tissues. Further experiments were conducted in 2012, by the same group, using a radiolabeled 143 nm peptide-targeted liposome engineered to expose the CRPPR to bind the heart endothelium. This approach led to a more than 30-fold increased liposome density of CRPPR-targeted particles in the heart than in the skeletal muscle [38].

Another interesting experiment was performed in 2011 by Dvir et al. who designed a nanoparticulate system capable of targeting the heart after MI. This targeting was based on the overexpression of the angiotensin II type 1 (AT1) receptor in the infarcted heart. Thus, 142 nm fluorescent PEGylated liposomes were conjugated with a ligand specific to AT1. These nanoparticles were able to specifically target cardiac cells *in vitro*. Significantly, higher levels of targeted liposomes, in fact, were found in the infarcted heart after *in vivo* intravenous injection at days 1, 4, and 7 in a murine model of MI [39].

As previously described, several cell types are involved in the postinfarction wound-healing process that can be used as targets for nanocarriers development. Harel-Adar et al. in 2010 reported of a new strategy for the modulation of cardiac macrophages to a reparative state based on the use of phosphatidylserine- (PS-) presenting liposomes intravenously administered to mimic the anti-inflammatory effects of apoptotic cells. In a rat model of MI, effective targeting was demonstrated by MRI and the efficacy of the approach was verified by the increase, *in vivo* and *in vitro*, of anti-inflammatory cytokines and the decrease of proinflammatory ones with a significant reduction in scar formation and cardiac remodeling [40].

## 4. The Stem Cell Therapy Approach

The human heart has a very limited regeneration ability [4].

In the attempt to regenerate functional CMs following MI, several approaches have been developed in recent years, ranging from stimulation of the intrinsic proliferative capacity of resident CMs to the enhancement of resident or not resident (tissue grafts) cardiac progenitor cell differentiation [41–47].

The stimulation of cell cycle resumption by mature CMs has been proposed as a feasible strategy in heart regeneration. Unlike lower vertebrates, in which the heart of adult individuals still retains some regenerative capabilities due to proliferation and differentiation of resident cells, the mammalian heart loses every regenerative capacity beyond the neonatal period, coinciding with CM cell cycle arrest. In rodents, for example, CMs reach a cell cycle arrest in the first postnatal week [48].

With the aim to reactivate CM proliferation, investigators tried to modulate cell cycle checkpoints by the stimulation of specific signalling pathways required to sustain proliferation and differentiation of CMs during development, such as the neuregulin1-ErB2/B4 [49]. Recombinant human neoregulin1 (NRG1) has entered clinical trials for heart failure, and parenteral administration of NRG1 in patients seems to improve cardiac function up to three months [50], but the extent to which CM proliferation contributes to these beneficial effects is still unknown.

Another strategy exploited for CM cell cycle restarting has been the forced expression of specific cell cycle regulators, such as cyclins and CDCs, whose downregulation accounts for proliferation arrest [51]. In some cases, this strategy successfully stimulated cardiomyocyte proliferation but caused extensive, lethal cardiac pathology [41].

Coordinated activation of promitogenic gene programs may have greater success than overexpression of individual cell cycle regulators. This goal has been achieved through the redeployment of developmental regulatory circuits, as those depending on Hippo signalling pathway, a highly conserved pathway that governs cell proliferation and organ size [52]. Mutations in the Hippo pathway that enhance the transcriptional activity of its main effector, YAP1, stimulated-foetal cardiomyocyte proliferation and caused profound cardiac overgrowth [53].

Finally, microRNAs (miRNAs) also offered a means to activate a mitogenic program in CMs, being attractive therapeutic targets because of their small size and easy deliverability. The use of miR-302-367 mimics, for example, promoted cardiac regeneration in MI murine models [54].

Although promising, some concerns arise from these studies towards clinical translation. While *in vitro* and small animal testing of this strategy are encouraging, clinical trials may be hindered by the risk of promiscuous or excessive cell replication, resulting in tumorigenesis [55].

Other investigators have suggested the transplantation of CM progenitor cells derived from different cell types as an alternative strategy for cardiac regeneration. Both the use of progenitor cells originating from pluripotent stem cells (i.e., embryonic stem cells [ESCs] or induced pluripotent stem cells [iPSCs]) [44–46] or those arising from adult progenitor cells located in the heart (known as resident cardiac progenitor cells—CPCs) or in noncardiac sites (nonresident CPCs), such as bone marrow- (BM-) derived CPCs [43, 56], have been tested as a source of transplantable cells. To date, more than 100 randomized phase I/II clinical trials have examined the therapeutic utility of BM-derived cells [56–59], but despite the safety profile, these strategies allowed only a marginal improvement of cardiac function, probably due to the low engraftment rate of transplanted cells and the few number of functional CMs derived from transplanted progenitors, resulting in poor beneficial effects [41].

Undifferentiated ESCs can be excluded from pluripotent cell-based therapies, as their injection into immunocompatible host hearts is related to teratoma formation [60], whereas injection of differentiated, murine or human ESC-derived CMs yielded stable grafts that improved rodent heart function [61, 62]. Interestingly, codelivery of paracrine factors and bioengineered microenvironments enhanced maturation of ESC-derived CMs [63]. Nevertheless, these CMs appeared immature, which reduced their efficacy due to arrhythmogenesis [64, 65].

The discovery of iPSCs by Takahashi and Yamanaka in 2006 [66] has changed the field of cardiac regenerative medicine, unveiling a new approach to heart regeneration. Since then, conversion of one differentiated cell type into another, without proceeding through a pluripotent intermediate, the so-called “direct reprogramming,” was reported for different cell types including CMs [67].

This furthered for searching factors that could drive the transdifferentiation of the abundant population of CFs found in the scar of MI zones, into therapeutically suitable cells, such as CMs. At present, combinations of transcription factors (TFs), miRNAs, and other agents have been tested for their ability to guide the transdifferentiation of fibroblasts into induced CMs (iCMs), both *in vitro* and *in vivo* [68]. Preliminary results of *in vivo* transdifferentiation of CFs showed encouraging improvements in MI rodent models [69–71]. Now, this promising strategy must be translated into the clinic for human cardiac tissue regeneration, but the use of integrative viruses, frequently adopted in many direct reprogramming approaches, generates concerns, mostly related to their association with the risk of oncogenesis and genomic disruption. As such, the development of optimized, nonintegrating methods for direct reprogramming will be crucial to begin clinical translation.

## 5. The Mechanism of Cellular Reprogramming

The feasibility of directly converting one cell type into another was first demonstrated in 1987, when it was shown that MyoD overexpression alone was sufficient to convert mouse fibroblasts into myoblasts [72]. Since then, cellular reprogramming has been shown to be an epigenetic process, which requires the re-expression of gene patterns that have been developmentally silenced and, as such, are found in closed chromatin regions [73]. The transition between different cellular states represents the final output of complex interactions among signalling pathways, TFs, and epigenetic regulators. For these reasons, reprogramming can be achieved with a combination of factors when no single factor would suffice [66–68].

The epigenetic remodelling related to the reprogramming of somatic cells to pluripotency has been extensively characterized [74, 75]. These results, together with natural examples of transdifferentiation observed in vertebrates [76] and temporal chromatin profiles along ESC differentiation into cardiomyocyte [77], provided further insight on identifying targeting molecules that can be modulated to drive lineage conversion.

Such evidence implies that TFs used for reprogramming need to have the ability to engage their target sites on nucleosomal DNA to open the chromatin. Actually, many TFs able to induce reprogramming are “pioneer factors,” differing from conventional TFs in their mechanism of action as they can access tightly packed chromatin structures and induce chromatin-remodelling events, allowing the subsequent binding of additional TFs or epigenetic remodelling enzymes [67]. Several studies have shown that chromatin decondensation by pioneer TFs progressively occurs during cell division and in turn exposes specific gene promoters in the DNA to which different TFs can now directly bind in trans, with chromatin-remodelling proteins that can either facilitate or hinder lineage conversion [73–74].

Small molecules that target enzymes involved in epigenetic modifications, such as DNA methyltransferases and histone deacetylase inhibitor, increase the efficiency of cellular reprogramming and sometimes can even functionally replace ectopic expression of certain TFs (as will be discussed later). Nevertheless, it remains largely unknown how the manipulation of universal epigenetic regulators activates the core gene regulatory network specific to the target cell type. Understanding these interactions will facilitate the identification of proper epigenetic regulators promoting lineage reprogramming.

**Figure 2:**
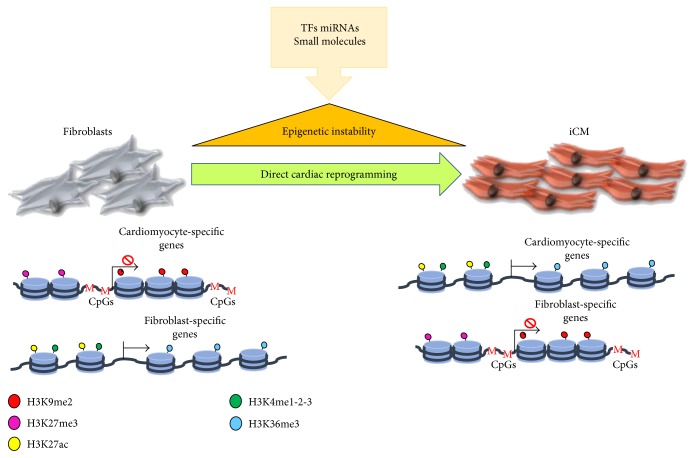
The mechanism of cellular reprogramming requires the re-expression of pattern of genes that have been developmentally silenced and, as such, are found in closed chromatin regions, and the silencing of the somatic active genes, which are presented, instead, in an active chromatin conformation. TFs, miRNAs, and epigenetic regulators allow the switch.

Recently, the epigenetic dynamics accompanying direct cardiac reprogramming by TFs have been investigated [78], revealing an early repatterning of H3K27me3 and H3K4me3 at cardiac loci and late alterations at fibroblast loci (Figure 2). These changes in histone pattern dynamics are accompanied by activation of the cardiac program and a progressive suppression of the fibroblast fate.

Similarly, Dal-Pra et al. demonstrated that H3K27 demethylation is required for the induction of cardiac gene expression during reprogramming induced by a microRNA cocktail [79]. Also, the timing of histone methyltransferase inhibition is crucial for its effect on reprogramming. Late inhibition of the methyltransferase G9a, which catalyses H3K9me1/2, increases reprogramming efficiency [80]. Conversely, pretreatment of fibroblasts with the G9a inhibitor reduced reprogramming efficiency [81], demonstrating that drug administration at only specific time frames is sufficient to promote an increase in reprogrammed cells and that inhibition at other times resulted in no effect or a decrease in reprogramming efficiency [80].

## 6. Direct Cardiac Reprogramming: Booster and Barriers

Efforts to select the perfect combination of factors for direct lineage conversion have been built on decades of developmental biology research. Numerous studies in model organisms have identified growth factors, TFs, and miRNAs controlling cell fate during embryonic development, as relevant drivers of embryologic cardiac differentiation. To date, laboratories worldwide reported different combinations of factors capable of engineering cell fate to specifically obtain cardiomyocytes both *in vitro* and *in vivo* [reviewed in 68].

The first attempt to directly reprogram murine fibroblasts into iCMs consisted of retroviral delivery of three crucial cardiac TFs: *Tbx5*, *Mef2c*, and *Gata4* (GMT) [82]. This approach allowed the direct conversion of fibroblasts into cardiomyocytes without passing through a mesodermal or cardiac progenitor stage. Subsequently, other groups have reported the generation of iCM using alternative sets of reprogramming factors and extended these results to human fibroblasts [70, 71, 82].

MicroRNAs have also been used to enhance cardiac reprogramming, such as the muscle-specific miRNAs miR-1 and miR-133 [83–85]. Jayawardena et al. demonstrated that a combination of four miRNAs (miR-1, miR-133, miR-208, and miR-499) converted mouse fibroblasts to iCM in the absence of any exogenous transcription factors [86]. This was also the first study reporting that the conversion efficiency may be improved by JAK inhibitor I [87]. Similarly, inhibiting TGF*β* signalling [81, 88] or the epigenetic regulator Bmi1 [89] appeared to increase conversion efficiency.

The TGF*β* superfamily is known to influence several cellular functions, including embryonic differentiation, depending on the cellular context [90]. The superfamily includes the TGF*β* ligands, activins, nodal, GDFs, and BMPs, which signal through specific transmembrane receptors [90]. The specific inhibition of the activin and nodal receptors, Alk4 and Alk7, and the TGF*β* receptor, Alk5, avoids Smad2/Smad3 phosphorylation and the subsequent initiation of downstream signalling [90]. TGF*β* signalling inhibition probably increases iCM generation by the depression of fibroblast gene expression programs, but the precise mechanism of action remains not completely understood.

Conversely, Fgf and Vegf signalling stimulation by Akt activation, together with GMT transduction, greatly increased the yield of beating cardiomyocytes particularly in mouse embryonic fibroblasts [91], as well as the overexpression of Akt1 [92].

Ding and colleagues added a new piece of knowledge to the field, achieving successful fibroblasts conversion into iCM using a single factor (Oct4) and a chemical cocktail comprising a TGF*β* inhibitor (SB431542) in combination with a GSK3 inhibitor (CHIR99021), an inhibitor of lysine-specific demethylase 1 (Parnate) and a cAMP pathway activator (forskolin) [93].

**Figure 3:**
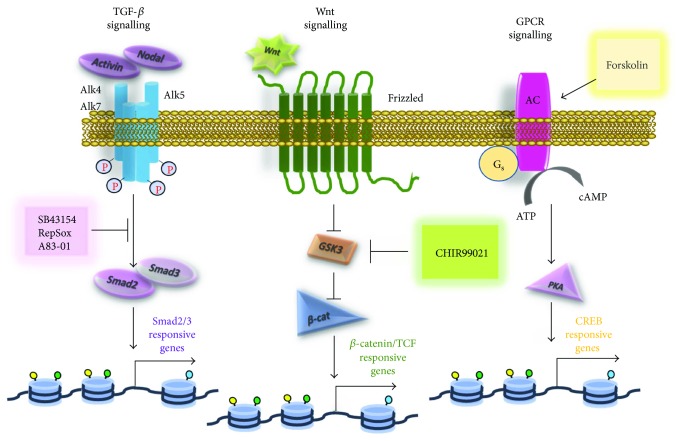
The main pathways modulated during direct cardiac reprogramming and the compounds involved.

The compound CHIR99021, by inhibition of GSK3b, activates the canonical Wnt pathway, which refers to *β*-catenin-driven pathway that is involved in various stages of embryonic mesoderm differentiation [94, 95]. As such, canonical Wnt signalling is indispensable during early stages of *in vitro* cardiogenic differentiation of mouse ESCs, in which Flk1+ mesodermal precursors do not emerge when the pathway is suppressed [94]. Parnate may be considered an epigenetic regulator, as it increases H3K4 methylation, thus enhancing the initial epigenetic activation of fibroblasts, whereas forskolin promotes the generation of intracellular cAMP and may facilitate gene expression via CREB-dependent mechanisms (Figure 3).

Together, these studies provided the proof-of-concept that fibroblasts can be directly reprogrammed into cardiomyocytes by modulation of combined signalling pathways.

While most of the TF-based approaches have used integrative retroviral or lentiviral vectors to deliver reprogramming genes to target cells, other investigators have tried to use small molecules alone to induce transdifferentiation via nongenetic strategies, providing a remarkable foundation for pharmacological interventions [96].

Small molecules represent an excellent tool for direct reprogramming, presenting several advantages over traditional methods: they may be permeable, easily synthesizable, and cheaper. Moreover, their effect can be fine-tuned by modulating their concentration and combination.

However, the identification of small molecules able to completely replace exogenous TFs remains a major challenge.

Recently, Fu et al. showed that a solely chemical cocktail comprising CHIR99021, RepSox (a TGF*β*R1 inhibitor), forskolin, and valproic acid (VPA—an HDAC inhibitor) could induce beating clusters of cardiac cells from mouse fibroblasts [97]. A chemical approach, based on the combination of nine compounds in part overlapping the cocktails used for reprogramming of mouse fibroblasts, also efficiently converted human fibroblasts to iCM. At both transcriptomic and epigenetic levels, these human iCMs resembled human cardiomyocytes. The authors observed enrichment of H3K4me3 as well as H3K27ac and decrease of H3K27me3 on a cohort of heart developmental genes [98].

This very thriving research activity, while proving effective in confirming the overall concept of direct reprogramming and its achievements, still faces the limitation of a poor yield of the whole process. In particular, in the pursuit of an effective cardiac regeneration, both the quantitative and qualitative yield are critical issues and lack of their accomplishment definitively compromises any further reasonable translational perspectives.

In this view, the field of nanotechnologies opens new scenarios to tailor the whole reprogramming to the desired target.

## 7. Nanotechnology and Direct Cardiac Reprogramming for Cardiac Regeneration

A possible synergy between the fields of cardiac direct reprogramming and nanotechnology is probably more than just a guess. Indeed, this interaction seems to be exploitable to facilitate the *in vivo* translation of *in vitro* acquired knowledge. There are several unmet needs to be addressed before cardiac direct reprogramming could be safely and effectively evaluated *in vivo*. To our opinion, these issues are mainly related to (i) the characterization of the most suitable cardiac resident candidate cell for direct reprogramming, (ii) the possibility of targeting the injured tissue with the appropriate amount of reprogramming factors without harming healthy cells, and (iii) the safe achievement of a functional integration of regenerated cells *in vivo*.

The candidate cell type to be reprogrammed represents the first issue to be addressed. These cells have to be abundant enough in the heart and easily accessible in the infarcted area. More importantly, the candidate cells need to be thoroughly characterized in order to be selectively and specifically targeted by nanocarriers. Following myocardial infarction, as previously described, an extensive remodelling process takes place in the damaged tissue. This process is mostly sustained by the activation of cardiac resident fibroblasts, whose biology is becoming the focus of research because of several knowledge gaps in their function [99, 100]. Due to their characteristics, these cells may represent a valid candidate for cardiac direct reprogramming and in turn deserve further studies in order to be better characterized as selected targets for nanocarrier delivery. On this ground, the most suitable reprogramming cocktail seems the one based on the use of chemical compounds which offers, at least theoretically, an acceptable safety profile as it does not imply genetic manipulations. However, to be fully exploited, the potential of chemical compounds in cardiac reprogramming requires a better tuning and handling with tailored delivery strategies, allowing compounds administration at a constant and defined rate to selected target cells. To this aim, research is needed to engineer more suitable nanocarriers starting from the identification of the more appropriate material of which nanocarriers can be made. In addition to the traditional nanocarriers, biocompatible polymers can be used to design several different types of nanocarriers tailored on the desired target. Moreover, *in vivo* translation will necessarily require a very selective active targeting strategy, which can only derive from the deep knowledge of candidate cells. The achievement of reprogrammed cardiomyocytes with defined functional phenotypes can, ultimately, be the consequence of the refined choice of the candidate cell and the appropriate chemical compound cocktail delivered in an adequate nanocarrier.

## 8. Conclusions

Although some key factors for cardiac reprogramming have been identified, a deeper knowledge of signalling networks that determine cell fate is required to select new combinations of small molecules capable of governing direct cardiac reprogramming in a more efficient way.

The finding that the removal of epigenetic barriers increases the efficiency of cardiac reprogramming suggests the type of interventions that can be implemented to achieve significant improvements.

Nevertheless, to achieve the full potential for chemical reprogramming *in vivo* and to project cardiac reprogramming to a preclinical stage, it will be necessary to develop innovative delivery strategies. Advances in nanotechnology will provide a tool to efficiently deliver compounds at a defined and sustained rate.
